# Integrated Immunomodulatory Mechanisms through which Long-Chain *n*-3 Polyunsaturated Fatty Acids Attenuate Obese Adipose Tissue Dysfunction

**DOI:** 10.3390/nu9121289

**Published:** 2017-11-27

**Authors:** Danyelle M. Liddle, Amber L. Hutchinson, Hannah R. Wellings, Krista A. Power, Lindsay E. Robinson, Jennifer M. Monk

**Affiliations:** 1 Department of Human Health and Nutritional Sciences, University of Guelph, Guelph, ON N1G 2W1, Canada; dliddle@uoguelph.ca (D.M.L.); hutchina@uoguelph.ca (A.L.H.); hwelling@uoguelph.ca (H.R.W.); 2 School of Nutrition Sciences, University of Ottawa, Ottawa, ON K1N 6N5, Canada; Krista.Power@uottawa.ca

**Keywords:** *n*-3 polyunsaturated fatty acids, obesity, adipose tissue, immune cell infiltration, inflammation, adipokines, T cells, macrophages

## Abstract

Obesity is a global health concern with rising prevalence that increases the risk of developing other chronic diseases. A causal link connecting overnutrition, the development of obesity and obesity-associated co-morbidities is visceral adipose tissue (AT) dysfunction, characterized by changes in the cellularity of various immune cell populations, altered production of inflammatory adipokines that sustain a chronic state of low-grade inflammation and, ultimately, dysregulated AT metabolic function. Therefore, dietary intervention strategies aimed to halt the progression of obese AT dysfunction through any of the aforementioned processes represent an important active area of research. In this connection, fish oil-derived dietary long-chain *n*-3 polyunsaturated fatty acids (PUFA) in the form of eicosapentaenoic acid (EPA) and docosahexaenoic acid (DHA) have been demonstrated to attenuate obese AT dysfunction through multiple mechanisms, ultimately affecting AT immune cellularity and function, adipokine production, and metabolic signaling pathways, all of which will be discussed herein.

## 1. Introduction

The prevalence of overweight and obese individuals within the global population (consisting of both developing and developed countries) has steadily increased over the past 30 years [1,2]. As defined by body mass index (BMI), this increased prevalence of overweight (25–29.9 kg/m^2^) and obese (>30 kg/m^2^) individuals represents a global health epidemic [1] that is associated with the development of a myriad of other chronic conditions and diseases, including but not limited to insulin resistance (IR), hypertension, and dyslipidemia (collectively referred to as Metabolic Syndrome [3]), cardiovascular disease (CVD), type 2 diabetes (T2D), stroke, certain cancer types, liver and gall bladder disease, osteoarthritis, sleep apnea, and gynecological conditions [1,4]. Furthermore, the consequences of obesity extend beyond these physical comorbidities to include an increased risk of various psychological and psychosocial conditions [5,6,7,8].

**Figure 1:**
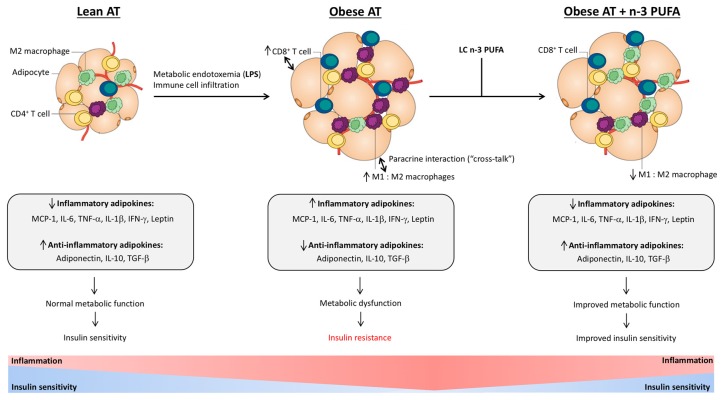
Lean adipose tissue (AT) maintains an anti-inflammatory and insulin-sensitive tissue microenvironment through the secretion of anti-inflammatory adipokines (adiponectin, interleukin (IL)-10 and transforming growth factor (TGF)-β) and is populated by immune cells including M2-polarized macrophages and cluster of differentiation (CD)4^+^ regulatory T (Treg) cells. However, during the development of obesity, the population of AT immune cells shifts, demonstrated by an increase in M1 macrophage accumulation, CD4^+^ T helper 1 cells, and CD8^+^ T cells, which secrete (along with adipocytes) inflammatory adipokines, monocyte chemoattractant protein (MCP)-1, interleukin (IL)-6, tumor necrosis factor (TNF)-α, IL-1β, interferon (IFN)-γ and leptin, thus promoting an inflammatory AT microenvironment. Increased circulating lipopolysaccharide (LPS; i.e., metabolic endotoxemia) further promotes the secretion of inflammatory adipokines from adipocytes and immune cells in obese AT, promoting the paracrine interactions (“cross-talk”) between obese AT-infiltrated immune cells and resident adipocytes, leading to the development of metabolic dysfunction and insulin resistance (IR). Supplementation with long-chain (LC) *n*-3 polyunsaturated fatty acids (PUFA), eicosapentaenoic acid (EPA) and docosahexaenoic acid (DHA), shifts the obese AT immune cell population towards a less inflammatory phenotype as suggested by the reduced M1:M2 macrophage ratio and increased anti-inflammatory adipokine production (adiponectin, IL-10, TGF-β), which improves metabolic function and insulin sensitivity.

In obesity, visceral adipose tissue (AT) exhibits a dysfunctional phenotype in comparison to that derived from lean individuals [9,10,11,12,13]. Functionally, AT is the primary storage site for excess energy in the form of triacylglycerol (TAG), but also functions as an endocrine organ that secretes signaling proteins, collectively termed adipokines (or cytokines, when not of AT origin), which influence systemic metabolism and immune function [11,14,15,16]. AT is composed of adipocytes and a non-adipocyte stromal vascular fraction (SVF), comprised of innate and adaptive immune cells, which are collectively engaged in the maintenance of AT insulin sensitivity (reviewed in [15,17,18,19,20]). However, overnutrition induces changes in the number and activity of AT immune cell populations [12,13,21,22,23,24,25] and the consequent paracrine interactions, or cross-talk, between those AT-infiltrated immune cells and resident adipocytes leads to increased production of inflammatory adipokines (e.g., leptin, monocyte chemoattractant protein (MCP)-1/chemokine (C-C motif) ligand 2 (CCL2), interleukin (IL)-6, tumor necrosis factor (TNF)-α and IL-1β) and decreased production of anti-inflammatory and insulin-sensitizing adipokines (e.g., adiponectin, IL-10) (reviewed here and in [19]) (shown in Figure 1). Accordingly, obese AT is characterized by chronic low-grade inflammation, which disrupts its normal functions and thus contributes to the development of Metabolic Syndrome [14]. As such, it is suggested that AT inflammation mechanistically links obesity to the development of CVD [26] and T2D [27].

**Figure 2:**
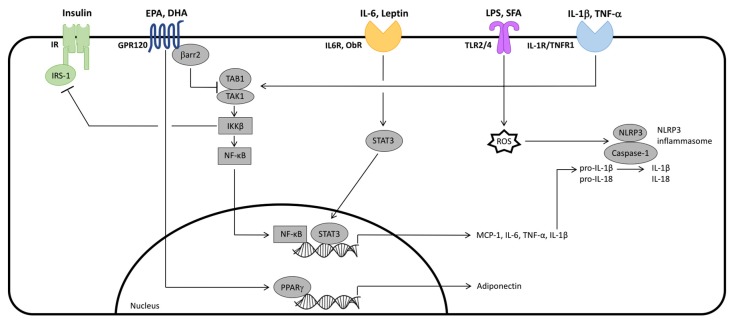
Integration of selected cell signaling mechanisms regulated by long-chain (LC) *n*-3 polyunsaturated fatty acids, eicosapentaenoic acid (EPA) and docosahexaenoic acid (DHA), and inflammatory adipokines in obese adipose tissue (AT). EPA and DHA stimulate G-protein coupled receptor (GPR)120, which promotes the association between β-arrestin 2 (βarr2) and GPR120. This complex internalizes, allowing βarr2 to bind transforming growth factor-β activated kinase (TAK)1 binding protein (TAB)1, which inhibits TAK1/TAB1 binding and subsequent nuclear factor κ-light-chain-enhancer of activated B cells (NF-κΒ) activation. TAK1/TAB1 binding also leads to the inhibition of insulin signaling. Lipopolysaccharide (LPS) and saturated fatty acids (SFA) stimulate Toll-like receptor (TLR)2 and TLR4, and interleukin (IL)-1β and tumor necrosis factor (TNF)-α stimulate IL-1 Receptor (IL-1R) and TNF Receptor 1 (TNFR1), respectively, all of which promote TAK1/TAB1 binding and subsequent NF-κΒ activation. NF-κΒ (as well as IL-6 and leptin-induced signal transducer and activator of transcription (STAT)3) regulate inflammatory adipokine gene transcription. Among them, the immature protein form of IL-1β undergoes further processing to the mature form by the caspase-1 subunit of the nucleotide-binding oligomerization domain-like receptor, pyrin domain containing (NLRP)3 inflammasome, activated by TLR2/4-induced reactive oxygen species (ROS) accumulation. EPA and DHA signaling also promotes peroxisome proliferator-activated receptor (PPAR)γ activation and subsequent adiponectin gene expression.

Nutritional strategies for the prevention and treatment of AT dysfunction are of increasing importance given the prevalence of obesity [1,2]. As discussed throughout this review, dietary fatty acids, namely the fish oil (FO)-derived long-chain (LC) omega-3 (*n*-3) polyunsaturated fatty acids (PUFA), eicosapentaenoic acid (20:5*n*-3, EPA) and docosahexaenoic acid (22:6*n*-3, DHA), have been extensively studied by our research group and others as anti-inflammatory nutrients (the broader clinical implications of which have been reviewed in [28,29]) (shown in Figure 2). Investigation into the mechanisms underlying the *n*-3 PUFA-mediated attenuation of aspects of the obese inflammatory phenotype and/or associated metabolic dysfunction rely on rodent high-fat diet (HFD)-induced and genetic obesity models, whereas such evidence still requires confirmation in humans. Furthermore, despite efforts by our research group [30,31] and others [28,32,33], determinations of the minimum effective dosage of *n*-3 PUFA to modulate critical aspects of the obese human phenotype, namely systemic inflammation, are limited by the diversity amongst study designs (e.g., participant characteristics) and *n*-3 PUFA interventions (e.g., source and duration). Nevertheless, evidence from in vitro and rodent in vivo studies suggests that EPA and DHA can decrease inflammatory adipokine production in adipocytes [34,35,36,37,38,39,40,41,42,43]. Further, in an in vitro co-culture model of obese AT, we have shown that LC *n*-3 PUFA reduce the inflammatory cross-talk between adipocytes and (i) macrophages [44,45]; (ii) cluster of differentiation (CD)8^+^ T cells [46,47,48] and (iii) (CD)4^+^ T cells [49] via, at least in part, downregulation of inflammatory adipokine synthesis and secretion. As discussed herein, multiple factors contribute to the development of AT dysfunction in obesity, but the focus of this review will include the changes in AT immune cellularity, dysregulation of adipokine secretion, and inflammatory signaling mechanisms in obese AT, while identifying potential targets for *n*-3 PUFA intervention to mitigate the ensuing metabolic consequences of obesity.

## 2. Adipose Tissue Function and Obesity-Associated Dysfunction

### 2.1. Healthy Adipose Tissue Function

AT plays a fundamental role in the regulation of whole-body metabolic homeostasis, serving as both an energy storage depot and an active endocrine organ [15,16]. Adipocytes comprise approximately 90% of AT volume, but only 20–40% of the overall cellular content [21,22,23,50]. Hence, AT is a heterogeneous tissue composed of mature adipocytes and a non-adipocyte cells that comprise the SVF, which includes adipocyte progenitor cells and immune cell populations (e.g., macrophages, dendritic cells, natural killer cells, B cells and T cells (CD8^+^ and CD4^+^ T cell subsets) [15,20]. Functionally, within AT, adipocytes share several common features with immune cell populations, including expression of the innate pattern recognition receptors (PRR), nucleotide-binding oligomerization domain (NOD)-like receptors (NLR) and Toll-like receptors (TLR)2 and TLR4 [51,52,53]. In response to, for example, certain ligands (e.g., lipopolysaccharide (LPS) or adipokines), nutrient and/or oxygen status within the AT microenvironment, PRR signaling regulates the synthesis and secretion of a wide range of adipokines (shown in Figure 2), which in turn influence local and systemic immune function, and fatty acid and glucose homeostasis (reviewed in [54]).

Mature adipocytes are the primary energy storage cell type within AT, and as a dynamic tissue, AT undergoes remodeling to adjust its storage capacity to meet the energy storage demand (reviewed in [16,55,56]). During periods of excess energy intake (i.e., positive energy balance), insulin stimulates adipocytes to store free fatty acids (FFA) in the form of neutral TAG through their esterification to glycerol [16]. Likewise, adipocytes manage insulin-stimulated glucose uptake and utilization as a substrate for *de novo* lipogenesis; and finally mobilize these TAG stores via lipolysis for transport back into the circulation during energy deficit [16]. Intracellular TAG accumulation promotes adipocyte hypertrophy, which reduces the blood flow and delivery of oxygen per unit of adipocyte surface [55,56]. Hypoxic adipocytes become necrotic and secrete inflammatory adipokines that recruit immune cells, via chemotaxis, to phagocytose necrotic cell debris and stimulate local angiogenesis [55,56,57,58]. Dead adipocytes are eventually replaced with new, smaller adipocytes, ultimately increasing the capacity of AT to store excess energy [55,56].

### 2.2. Adipose Tissue Dysfunction in Obesity

AT is distributed throughout the body in subcutaneous and visceral depots; the former is generally considered to be a safer long-term energy storage depot in comparison to visceral AT depots, which increase in size in obesity and are associated with the development of metabolic complications [59,60,61,62]. To compensate for the continuous supply of FFA during overnutrition (positive energy balance), TAG accumulate within visceral AT adipocytes, thus inducing adipocyte hypertrophy [16]. Large adipocytes are characterized by decreased sensitivity to insulin and its anti-lipolytic effects, as well as dysregulated adipokine synthesis and secretion [60,63]. Eventually, adipocyte dysfunction leads to and is exacerbated by a ‘spillover’ of FFA, primarily of the saturated fatty acid (SFA) class (e.g., palmitic acid (16:0, PA)) [16], which may act in an autocrine or paracrine manner as ligands for inflammatory TLR2/4 signaling (shown in Figure 2). Specifically, SFA-induced TLR2/4 signaling induces a network of intracellular responses that further contribute to adipokine dysregulation and sustained chronic low-grade inflammation, including activation of the nuclear factor κ-light-chain-enhancer of activated B cells (NF-κΒ) transcription factor and the NLR, pyrin domain containing (NLRP)3 inflammasome [54,64,65,66,67,68,69,70,71,72,73,74,75] (discussed in Section 6). Ultimately, the autocrine and paracrine feedforward consequences of adipocyte dysfunction (i.e., adipokine dysregulation, FFA release) lead to whole AT dysfunction (reviewed here and in [16]). Specifically, dysfunctional AT cannot meet the ongoing demand for increased energy storage capacity during overnutrition and, coupled with its increased lipolytic activity, dysfunctional AT gives rise to chronically elevated circulating FFA levels [9,10] as reported in obese and T2D patients [76,77,78]. The spillover of FFA is delivered to ectopic tissues, including the liver and skeletal muscle, wherein the accumulation of lipid intermediates (e.g., diacylglycerol and ceramides) together with the abundance of inflammatory stimuli (e.g., inflammatory adipokines) ultimately impairs insulin signaling (reviewed in [15]), thereby causally linking AT dysfunction to systemic IR.

### 2.3. Adipokine Dysregulation in Obese Adipose Tissue

Adipokine synthesis and secretion from adipocytes and SVF cells is essential for normal AT function as a central regulator of systemic immunity and metabolism (reviewed in [15,16,79]). However, the constant demand to increase energy storage capacity during overnutrition leads to AT dysfunction, increased FFA release [63], and dysregulation of adipokine synthesis and secretion [11,60,80]; a process that we and others have shown is exacerbated by cross-talk between adipocytes and various immune cell populations [44,45,46,47,48,65,81]. The adipokine profiles of obese subcutaneous and visceral AT depots differ wherein visceral AT is associated with the metabolic complications of obesity [60,80]. Ultimately, obese visceral AT is characterized by a state of chronic low-grade inflammation owing, in part, to increased secretion of inflammatory adipokines and decreased secretion of anti-inflammatory and insulin-sensitizing adipokines (reviewed in [15,16,79]) (shown in Figure 1; discussed in Section 5). While an increasing number of adipokines are implicated in the development of the obese phenotype, the focus of this review will include MCP-1, IL-6, TNF-α, IL-1β, leptin and adiponectin as the key mediators of AT inflammation and dysfunction in obesity.

### 2.4. Metabolic Endotoxemia Drives Adipose Tissue Dysfunction in Obesity

Evidence from rodent models suggests that, in obesity, AT dysfunction is driven, in part, by increased circulating bacterial components (e.g., LPS, peptidoglycan, flagellin) and metabolites (e.g., secondary bile acids) (as reviewed in [82]). Among them, LPS, a component of Gram-negative bacteria cell walls [83,84,85,86,87,88], is the most commonly studied bacteria-derived inflammatory stimulus considered in investigations into the mechanistic link between the gut microbiota and obesity-associated inflammation. Studies assessing the effect of *n*-3 PUFA supplementation on either the lean or obese microbiome are limited [89,90], and therefore are beyond the scope of this review. Obesity is a gut-associated disease wherein the intestinal microenvironment of lean versus obese individuals differs dramatically including a dysbiotic microbial community structure and activity [91,92,93,94,95,96,97,98,99] and impaired intestinal epithelial barrier function [100,101]. The impaired obese intestinal microenvironment contributes to critical aspects of the obese pathology, in part by increasing energy harvested from non-digestible food components and initiating intestinal barrier dysfunction leading to enhanced barrier permeability, microbial invasion, and AT and systemic inflammation and metabolic dysfunction [83,101,102,103]. Moreover, these obesity-associated changes within the human intestinal microenvironment can be recapitulated in animal HFD-induced obesity models [83,104,105,106]. Studies performed in germ-free (microbiota-free) mice colonized with the cecal content obtained from obese mice have demonstrated that the disease phenotype (inflammation and/or metabolic dysfunction) can be transferred to the germ-free recipients through microbial colonization, thereby suggesting that gut microbes can drive metabolic alterations within the host tissues that ultimately produce the disease phenotype [107,108]. An important feature of the impaired obese intestinal microenvironment that contributes to the obese phenotype is reduced intestinal epithelial barrier integrity (i.e., “leaky gut”), which promotes bacterial translocation across the epithelial barrier leading to the development of increased systemic inflammation (metabolic endotoxemia), driven by bacterial-derived LPS signaling [91,103]. LPS is a potent systemic inflammatory stimulus that is significantly elevated in the blood of obese individuals [101,109] and rodents [83,110], and increased circulating LPS levels are attributable to a decrease in epithelial barrier function and integrity [83,91,111]. Intestinal bacterial overgrowth during obesity increases LPS levels within the enteric cavity, leading to greater epithelial mucosal barrier damage, increased bacterial translocation into the host tissues and, ultimately, metabolic endotoxemia [112]. LPS is a ligand for TLR2/TLR4 (shown in Figure 2; discussed in Section 6.1) expressed on the surface of adipocytes and immune cells [52], which signals to initiate NF-κB activation and adipokine secretion, thereby perpetuating the obese low-grade inflammatory state and subsequent metabolic dysfunction, including AT and systemic IR [66,83,85,113,114,115,116] (shown in Figure 1). For instance, in healthy humans, acute LPS administration increased AT production and circulating levels of MCP-1, IL-6 and TNF-α prior to the development of systemic IR [85]. Similarly, metabolic endotoxemia induced in mice via continuous infusion of LPS increased visceral AT mass, immune cell (i.e., macrophage) infiltration, and inflammation, as well as fasting glycaemia and insulinemia to a similar extent as observed in HFD-fed mice [83]. Importantly, the metabolic consequences of both LPS-infusion (i.e., metabolic endotoxemia) and HFD feeding were blunted in TLR4-deficient mice [83,117]. Thus, LPS is an important factor in obesity-induced AT dysfunction and warrants inclusion in studies investigating strategies to modulate adipokine synthesis and secretion.

## 3. Dietary *n*-3 Polyunsaturated Fatty Acids as a Strategy to Modulate Adipose Tissue Dysfunction in Obesity

Strategies to modulate adipokine synthesis and secretion from AT are warranted to attenuate the chronic low-grade inflammation that causally links obesity to pathologies such as systemic IR, CVD and T2D [14,26,27,60,85,118]. Since AT is adept at responding to nutritional stimuli, dietary *n*-3 PUFA may provide such a strategy by regulating the activity of adipokine and immune cell receptors that are intrinsic to adipokine modulation in the obese state.

*n*-3 PUFA can act as stimuli for specific cell membrane-bound (e.g., G protein-coupled receptor (GPR)120, discussed in Section 6.2) or intracellular (e.g., peroxisome proliferator-activated receptor (PPAR)-γ) receptors to directly influence inflammatory adipokine production by adipocytes and immune cells within AT (reviewed herein and in [119,120]). Further, LC *n*-3 PUFA membrane enrichment influences membrane fluidity, formation of lipid rafts and subsequent signal transduction efficiency (discussed here and in [121,122,123]), or can serve as substrates for the synthesis of bioactive lipid mediators (i.e., eicosanoids), which can influence inflammatory signaling (reviewed in [29]).

## 4. Obese Adipose Tissue Immune Cells and Modulation by *n*-3 Polyunsaturated Fatty Acids

### 4.1. Altered Immune Cell Composition in Obese Adipose Tissue

In lean AT, various immune cells, such as M2-polarized macrophages (F4/80^+^, CD11b^+^, CD11c^−^) and CD4^+^ regulatory T (Treg) cells (CD4^+^, forkhead box P3 (FOXP3)^+^), are engaged in the maintenance of insulin sensitivity, partly through their secretion of anti-inflammatory adipokines (reviewed in [15,17,18,19,20]). However, overnutrition induces changes in the number and activity of visceral AT immune cell populations, which collectively direct development of the obese phenotype [12,13,21,22,23,24,25] (shown in Figure 1). For instance, obese AT is characterized by increased macrophage accumulation, a greater proportion of which are polarized to the M1 inflammatory phenotype (F4/80^+^ CD11b^+^ CD11c^+^; discussed in Section 4.2) [12,21,22,124,125,126,127]. Further, Treg cell abundance is significantly reduced in obese AT [22,23,128,129,130], as are CD4^+^ T helper (Th)2 cells [13,23]. Conversely, the proportion of infiltrating dendritic cells [131,132], B cells [133,134], NK cells [135,136,137], CD4^+^ Th1 cells [13,22,23,138,139], and CD8^+^ T cells [13,22,23] are reported to increase in obese AT.

As obesity progresses, AT-derived FFA and inflammatory adipokines act in a controlled autocrine, paracrine and endocrine manner to ultimately recruit and activate immune cells in an attempt to repair the AT dysfunction (reviewed in [15,17,18,19,20]). However, as the metabolic consequences of overnutrition persist, immune cell infiltration into obese AT becomes dysregulated [22,23,24,124,133,137,140]. Specifically, FFA and LPS serve as ligands for adipocyte and immune cell TLR2/4 signaling [52,66,68,83,141,142] (discussed in Section 6.1), which promote the synthesis and secretion of inflammatory and chemotactic adipokines via, in part, NF-κΒ and NLRP3 inflammasome activation [73,74,143,144] (discussed in Section 6.1 and Section 6.3, respectively). Further, our research group and others have shown that the gene expression and/or secretion of inflammatory adipokines increases with LPS stimulation in adipocytes [43,116,145,146,147] and immune cells alone [38,147,148,149], as well as in co-culture [45,46,47,48,49,81], suggesting that the cross-talk between obese AT-infiltrated immune cells and resident adipocytes yields a vicious cycle that leads to a local and systemic state of chronic low-grade inflammation and metabolic dysfunction [20]. In summary, though many other immune cell populations including dendritic cells [131,132], B cells [133,134], NK cells [135,136,137], neutrophils [150], eosinophils [151], and mast cells [152] play key roles in the maintenance of AT homeostasis and development of the obese AT phenotype, perhaps through direct or indirect modulation of resident AT macrophage responses (reviewed in [15,17,18,19,20]), this review will focus on adipokine-mediated cross-talk between adipocytes, macrophages and T cells (CD4^+^ and CD8^+^ subsets).

### 4.2. Obesity-Associated Changes in Adipose Tissue Macrophages

Among AT-infiltrating immune cell populations, macrophages have taken center stage as a hallmark of the obese AT phenotype since their degree of infiltration is associated with the progression of IR (reviewed in [75]). Specifically, the percentage of AT macrophages within the SVF increases by 20–30% in obese versus lean AT, in both humans and rodent obesity models [12,21,22,124,125,126,127]. AT macrophages cluster around necrotic adipocytes, forming crown-like structures (CLS) [153], which are observed more frequently in obese versus lean visceral AT [60].

There are two well-defined macrophage phenotypes that can be differentiated from circulating monocytes that infiltrate AT; however, macrophage phenotype is highly plastic in response to signals within the surrounding microenvironment [154,155], and thus these polarization states are not absolute and can vary along the M1–M2 spectrum [154]. These polarized macrophage subsets are referred to as M1, or ‘classically activated’, and M2, or ‘alternatively activated’, macrophages [154,156]. In obese AT, the macrophage population undergoes a phenotypic switch from the M2 phenotype (the dominant macrophage phenotype in lean AT) to the M1 phenotype, which functionally exacerbates AT inflammatory mediator production [12,21,22,124,125,126,127] (shown in Figure 1). The M1 macrophage phenotypic shift has been shown to occur when lipids are repartitioned from hypertrophic adipocytes to macrophages during obesity progression, wherein M1 macrophages form in response to lipotoxicity and resemble TAG droplet laden foam-cells [157].

Specially, M1 macrophages can be activated by the Th1 cytokine, interferon (IFN)-γ, or by TLR2/4 ligands such as gut-derived LPS or FFAs released from dysfunctional adipocytes [158,159,160,161]. The downstream effect of TLR2/4 signaling results in inflammatory adipokine secretion from M1 macrophages, yielding a paracrine communication loop with adipocytes and other immune cell populations that contribute to local AT, and ultimately systemic metabolic dysfunction [65,68]. Post-activation, M1 macrophages undergo a respiratory burst followed by production of reactive oxygen (ROS) and reactive nitrogen species (RNS) that promote microbicidal responses, antigen presentation via the major histocompatibility complex (MHC)II, and the secretion of adipokines that promote Th1 and/or Th17 cell-mediated responses [155,156].

Overactivation of M1 macrophages promote chronic inflammation [154], as these cells are characterized by increased lipid content, increased expression of anti-microbicidal inducible nitric oxide synthase (iNOS, observed in murine obese AT only), and secretion of inflammatory adipokines, namely IL-6, TNF-α and IL-1β, which impair adipocyte insulin signaling and promote lipolysis [12,69,75,124,126,154,157,162,163]. In this connection, M1 macrophages localize to CLS and macrophage-derived adipokines have been shown to perturb adipocyte insulin sensitivity [164,165] leading first to local AT IR and ultimately systemic IR [12,14,126,153,162,166,167]. M1 macrophages can be identified by increased cellular surface expression of integrin/complement receptors 3 (CD11b) and 4 (CD11c) [164] and this AT macrophage subset (F4/80^+^, CD11b^+^, CD11c^+^) has been shown to exhibit an enhanced inflammatory response when exposed to FFAs (i.e., TLR2/4 ligands), which is abrogated by TLR-4 antagonism [66]. Further, CD11c^+^ expressing macrophages appear to be a crucial contributor to obese AT dysfunction, as ablation of these cells results in reduced systemic inflammation and normalization of insulin sensitivity [168,169].

Conversely, M2 macrophages are characterized by the expression of scavenging receptors, mannose receptor (CD206) and macrophage galactose-type C-type lectin 1 (MGL1), and MHCII and co-stimulatory molecules [155]. Maturation of M2 macrophages, which exhibit anti-inflammatory and regulatory (i.e., tolerogenic) immune functions, is promoted by anti-inflammatory mediators such as IL-10, transforming growth factor (TGF)-β and glucocorticoids [156], and the ingestion of apoptotic cells [170]. Interestingly, macrophage polarization is not absolute and may shift throughout the development of obesity, or with weight loss interventions [171]. Therefore, macrophage populations exhibiting a mixed M1/M2 phenotype have been observed in obese AT in mice [171,172] and humans [12,167,173,174,175], wherein these cells express moderate levels of M1 (CD11c) and M2 (MGL1 or CD206) cell surface markers.

### 4.3. Influence of n-3 Polyunsaturated Fatty Acids on Obese Adipose Tissue Macrophages

Cross-talk between adipocytes and macrophages in obese AT represents a significant contributor to the obese AT inflammatory adipokine profile [65,69]. Multiple studies have demonstrated the effect of LC *n*-3 PUFA supplementation in HFD-induced rodent models of obesity, including reduced visceral AT M1 macrophage accumulation in favour of M2 macrophage polarization [42,176,177,178,179,180,181,182], which is associated with decreased inflammatory mediator production and increased systemic insulin sensitivity [176,177,179] (shown in Figure 1). Interestingly, these effects are similar to those reported in the HFD-fed *Fat-1* mouse, which is capable of synthesizing LC *n*-3 PUFA *de novo* [183]. In randomized controlled human interventions, *n*-3 PUFA supplementation has been shown to reduce the formation of CLS observed in abdominal AT biopsies, which was associated with reduced circulating MCP-1 levels [184]; whereas macrophage number and CLS abundance in subcutaneous abdominal AT biopsies were unaffected by *n*-3 PUFA supplementation [185], thereby highlighting the differential effects between subcutaneous and visceral AT depots.

Mechanistically, cell culture studies from our group and others’ have demonstrated the ability of LC *n*-3 PUFA to reduce macrophage M1 polarization while promoting M2 polarization status, and to inhibit inflammatory adipokine expression and/or secretion [35,38,44,165,184,186,187,188]. Furthermore, these effects have been shown to be dependent, at least in part, on various mediators and signaling pathways, including adiponectin [187] and PPARγ activation [186], although further study is required. Interestingly, *n*-3 PUFA-derived lipid mediators, namely resolvins and protectins, which exert anti-inflammatory and pro-resolving physiological functions [189,190], have also been shown to influence macrophage function and inflammatory mediator production by stimulating phagocytic activity, decreasing infiltration into CLS, and promoting their polarization towards the M2 phenotype [179,191,192,193,194]. Ultimately, decreased secretion of inflammatory adipokines from obese AT could serve to decrease the ongoing recruitment of macrophages and ensuing inflammatory adipocyte-macrophage cross-talk [35,38,44,165,184,186,187] that exacerbates chronic inflammation in obesity.

### 4.4. Obesity-Associated Changes in Adipose Tissue T Cells

Despite the macrophage-centric focus of AT immune cells, several studies have demonstrated the significant contribution of T cells in the development of the obese phenotype, in both humans [13,25,140,195,196] and rodent models [22,23,24,25,50,138,195,196,197,198,199] (shown in Figure 1). In fact, T cells have been shown to localize to CLS [138,195,196,197], suggesting a role in the cross-talk between adipocytes and macrophages. Further, although controversy exists [168] and human data is less clear, changes in T cell AT infiltration (increased by 0.5–5% of the SVF), activation and/or effector status has been shown to precede significant AT macrophage accumulation in diet-induced obese rodent models [22,23,24,196]. In humans, a positive correlation between AT T cell accumulation and the degree of adiposity has been reported, with greater accumulation in visceral AT depots compared to subcutaneous [13,140], and the proportion of activated (CD25^+^, CD69^+^) AT T cells is consistently higher in obese individuals compared to lean [25]. Importantly, depletion of visceral AT T cells in diet-induced obese mice improved AT inflammation and systemic insulin sensitivity in young but not adult mice [50], suggesting an early window of time during which T cell-mediated immune function may be controlled in obesity.

Controversy exists over which T cell subset is the first to exhibit changes in cellular abundance or activation in obese AT; CD4^+^ T cells [23,196], CD8^+^ T cells [197,200,201], or both [13,24,138,177,202,203]. Such discrepancies have yet to be explained, but one reason could be the different methods used to express and normalize T cell number within AT, such as percentage of total SVF cells, number of cells/g of AT and number of cells/AT depot [17].

### 4.5. Obesity-Associated Changes in Adipose Tissue CD4^+^ T Helper Cell Subsets

CD4^+^ T cells are divided into Th1, Th2, Th17 and Treg subtypes as defined by the expression and/or production of signature transcription factors and cytokines (reviewed in [204]). Accordingly, obese AT is characterized by an increased cellular abundance of the inflammatory Th1 cell subtype [23,139,199,205] and a decreased abundance of the non-inflammatory Treg subtype [23,201,206,207,208]. However, controversy exits with respect to the AT abundance and function of Th17 and Th2 cell subsets in lean and obese AT [13,23].

Th1 cells can be identified by T-box transcription factor (T-bet) expression and the secretion of inflammatory cytokines such as IFN-γ, whereas Th2 cells are identified by GATA-binding protein (GATA)3 transcription factor expression and secrete anti-inflammatory IL-4 and IL-13 (reviewed in [18,204]). Although Th1 and Th2 cells are present in equal proportions in the AT of lean rodent models, diet-induced obesity has been shown to induce a dramatic increase in the Th1 cellular number, with no change in Th2 cells [23,139]. Likewise, human AT T cells have been reported to exhibit a Th1 (IFN-γ-secreting) profile [13,140], whereas the number of AT Th2 cells inversely correlates with IR and circulating liver-derived C-reactive protein (CRP) [13], a clinical inflammatory biomarker [209,210]. Accordingly, adipocyte-derived leptin and FFA, both of which are increased in obesity, have been shown to enhance Th1 proliferation and/or IFN-γ production [211,212].

In general, Th1 cells participate in obese AT inflammation and promote IR, primarily owing to their secretion of IFN-γ (reviewed in [18]). IFN-γ has been shown to stimulate adipocytes to express T cell and macrophage chemoattractants, whereas systemic depletion of IFN-γ reduces AT macrophage accumulation, local inflammation, and improved systemic insulin sensitivity [138]. In contrast, however, the depletion of all mature lymphocytes in the recombination activating gene (*Rag*)1-deficient mouse did not protect against HFD-induced obese AT inflammation and systemic IR [23]. Interestingly, adoptive CD4^+^ T cell transfer reversed these aspects of the obese phenotype, but predominantly through the actions of Th2 cells as adoptive transfer of T cells from signal transducer and activator of transcription (STAT)6-deficient rodents, which have normal Th1 but impaired Th2 development, did not [23]. Altogether, these data suggest the significant and opposing roles of Th1 and Th2 cells in the development of the obese phenotype.

Th17 cells are identified by retinoic acid receptor-related orphan receptor (ROR)γτ expression and IL-17 secretion (reviewed in [18,204]), and have been shown to promote inflammatory responses in autoimmune diseases; however, their role in obese AT requires further investigation. Th17 cells are detectable in AT, but their cellular abundance is minimal and remained unchanged in obese mice versus lean [23]; although, controversy exists since a higher proportion of Th17 cells has been observed in the peripheral blood and/or subcutaneous AT of obese humans [212,213,214,215]. Interestingly, the major cellular source of AT IL-17 has been reported to be γδT cells rather than αβT cells, and IL-17-deficient mice are more susceptible to HFD-induced obesity, but remain insulin sensitive [216]. Accordingly, IL-17 has been shown to impair adipogenesis and adipocyte expression of genes involved in glucose and lipid metabolism [216], in addition to increasing the pre-adipocyte, adipocyte and whole AT expression of inflammatory adipokines [217]. Likewise, IL-17 production has been reported to correlate with the severity of T2D in humans [218].

Tregs, identified by FOXP3 expression [204], play an important role in self-tolerance and reduce inflammation by suppressing autoreactive T cells and M1 macrophages (reviewed in [219]). Accordingly, in AT, Tregs are found dispersed between adipocytes, but also in CLS in close contact with macrophages and CD8^+^ T cells [128]. Lean AT is highly enriched with Tregs, comprising 30–40% of all AT CD4^+^ T cells; a proportion that is significantly higher compared to both lymphoid and other non-lymphoid tissues [23,128,220,221]. However, the Treg cellular proportion of all AT CD4^+^ expressing T cells decreases during the development of obesity [22,23,128,206], yet controversy exists [222]. Nonetheless, Treg depletion experiments have demonstrated their significance in limiting the development of the obese phenotype, wherein rodent models of genetic and HFD-induced obesity exhibited increased AT inflammation and decreased systemic insulin sensitivity [128,223]. Likewise, in similar rodent models of obesity, AT Treg accumulation produced opposing effects, consistent with a reduced AT M1 macrophage content and increased IL-10-producing M2 macrophages [23,128,224].

### 4.6. Influence of n-3 Polyunsaturated Fatty Acids on Obese Adipose Tissue CD4^+^ T Helper Cell Subsets

To our knowledge, there is only one report on the effect of *n*-3 PUFA on CD4^+^ T cells in obese AT wherein *n*-3 PUFA supplementation did not affect mesenteric AT expression of CD4 or FOXP3 in HFD-induced obese mice [177]. However, dietary plant-derived *n*-3 PUFA (α-linolenic acid (18:3*n*-3, ALA)-enriched flaxseed) has been shown to reduce perirenal AT total CD3^+^ T cell abundance in obese leptin receptor-defective *fa*/*fa* Zucker rats [225]. The ability of *n*-3 PUFA to influence effector subset polarization and function of CD4^+^ T cells has been described in other experimental conditions or disease models, thereby providing the proof of concept that T cell effector subset polarization and function may be influenced within obese AT by *n*-3 PUFA, although further study is required.

Antigen-driven CD4^+^ T cell activation involves the formation of an immunological synapse and assembly of the signalsome protein complex, which is stabilized by the actin cytoskeleton [226,227,228]. Formation of the immunological synapse involves reorganization of nanoscale lipid rafts and signaling proteins [227,229,230] and T cell activation is suppressed with lipid raft disruption [231,232]. Importantly, *n*-3 PUFA have been shown to alter the stability and/or size of lipid rafts in CD4^+^ T cells [122,233,234,235,236] and suppress downstream cellular activation via mechanisms including (i) displacement of T cell activation-associated signaling proteins from detergent-resistant membrane fractions [122,235,237,238,239], (ii) IL-2 secretion [122,240,241,242], (iii) lymphoproliferation [235,243,244], and (iv) mitochondrial translocation [245]. With respect to specific CD4^+^ T cell subsets and Th1/Th2 balance, *n*-3 PUFA have been shown to influence both polarization and signature cytokine secretion in favor of the Th2 subset while concomitantly decreasing the activation of the Th1 subset [246,247,248]. Additionally, *n*-3 PUFA have been shown to reduce CD4^+^ T cell polarization of Th17 cells, in part through lipid raft-mediated disruption of IL-6 signaling [249,250], whereas there was no effect of *n*-3 PUFA on Treg polarization [249]. Interestingly, in a model of concurrent HFD-induced obesity and colitis, dietary supplementation of *n*-3 PUFA reduced visceral AT gene expression of the Th1 subset signature cytokine, IFN-γ, and the Th17 transcription factor, RORγτ, and signature cytokine, IL-17, with no effect on FOXP3 expression [178].

### 4.7. Obesity-Associated Changes in Adipose Tissue CD8^+^ T Cells

The primary function of CD8^+^ T cells, also known as cytotoxic T cells, is to kill infected cells by producing perforin, granzymes and inflammatory cytokines, namely IFN-γ (reviewed in [251]). The degree of splenic CD8^+^ T cell activation in vitro was demonstrated to be markedly increased when co-cultured with obese versus lean AT [22], which complements in vivo reports in humans and rodents wherein the proportion of activated CD8^+^ T cells is increased in the SVF of obese AT versus lean [22,23,24,25,50,196,198,252].

Functionally, the obese AT microenvironment does not influence the development or maintenance of memory CD8^+^ T cell responses that are primed either before or after obesity is established [253]. Specifically, obesity did not impact the maintenance or function of pre-existing memory CD8^+^ T cells (i.e., cell surface phenotypic markers, cytokine production and secondary expansion), nor the differentiation and maintenance of newly activated memory CD8^+^ T cell responses [253]. Conversely, CD8^+^ T cells have been shown to accumulate in AT and localize to CLS in advance of macrophage accumulation in obese humans and rodents [22,25,50,197]. Specifically, in HFD-fed mice, CD8^+^ T cells infiltrated visceral AT within two weeks and reached their peak cellular abundance of 10% of the SVF by 11 weeks. Conversely, however, macrophage AT infiltration was not observed until six weeks of HFD feeding, but continued to increase thereafter [22].

The significance of CD8^+^ T cells in macrophage-mediated metabolic dysfunction in obesity has been elegantly demonstrated by Nishimura and colleagues, both in vitro and in vivo [22]. Specifically, conditioned media collected from activated CD8^+^ T cells contained several chemotactic and inflammatory cytokines (e.g., MCP-1) that induced macrophage migration and activation. Interestingly, macrophage differentiation and proliferation were dependent upon the cross-talk between CD8^+^ T cells and obese AT, as neither CD8^+^ T cells nor AT alone exerted the same effect as co-culture. Further, CD8^+^ T cell depletion protected mice against HFD-induced obese visceral AT gene expression of inflammatory and macrophage chemotactic adipokines, which coincided with reduced M1 macrophage infiltration and CLS frequency without affecting the M2 macrophage fraction, as well as with improved systemic insulin sensitivity. However, adoptive transfer of CD8^+^ T cells into HFD-fed CD8^+^ T cell-deficient mice reversed this protection [22].

### 4.8. Influence of n-3 Polyunsaturated Fatty Acids on Obese Adipose Tissue CD8^+^ T Cells

Although mesenteric AT gene expression of CD8 was shown to be reduced with *n*-3 PUFA supplementation in HFD-induced obese mice [177], the effect of *n*-3 PUFA on obese AT CD8^+^ T cell abundance/tissue infiltration and function, namely cellular function and influence on the development of critical components of the obese AT phenotype, has not been determined in vivo. Interestingly, our group has shown the potential of LC *n*-3 PUFA to modulate obese AT function utilizing a co-culture model comprised of 3T3-L1 murine adipocytes and primary splenic CD8^+^ T cells purified from mice consuming a FO (i.e., LC *n*-3 PUFA-enriched) diet. The obese AT microenvironment was recapitulated using a co-culture cellular ratio of 10% CD8^+^ T cells to adipocytes, as described in obese AT by Nishimura and colleagues [22], which was stimulated with LPS at a concentration that mimics in vivo circulating levels in obesity [83,109,254]. LC *n*-3 PUFA-enriched co-cultures exhibited both an anti-inflammatory and anti-chemotactic secretory profile that consisted of reduced activation of inflammatory transcription factors (NF-κB and STAT3) and reduced secretion of both inflammatory and macrophage chemotactic adipokines, which was functionally confirmed by reduced macrophage chemotaxis [46] and polarization towards the M1 phenotype [47]. Furthermore, these findings were confirmed in separate studies utilizing the same co-culture model comprised of adipocytes and plant-derived *n*-3 PUFA (i.e., ALA)-enriched CD8^+^ T cells [255], and well as FO (i.e., LC *n*-3 PUFA)-enriched CD8^+^ T cells purified from obese mice [47]. Interestingly, in the latter model, we showed that LPS-stimulated adipocyte-CD8^+^ T cell inflammatory cross-talk and ensuing M1 macrophage polarization and adipocyte dysfunction are attenuated by TNF-α neutralization [47,48].

## 5. Adipose Tissue Inflammation in Obesity and Modulation by *n*-3 PUFA

### 5.1. Monocyte Chemoattractant Protein-1

MCP-1, also known as CCL2 in humans, is a potent chemoattractant that recruits circulating monocytes and macrophages to the site of inflammation (i.e., obese AT) via binding the cell membrane CCL2 receptor (CC2R) (reviewed in [256]). AT and circulating levels of MCP-1 are increased in obese humans [11,257], and in rodent models of HFD-induced and genetic obesity [70,258]. Visceral AT has been shown to secrete more MCP-1 than subcutaneous AT and, while controversy exists, adipocytes are reported to be the main cellular source [81,257,259] with increased MCP-1 production reported in adipocytes isolated from obese versus lean humans [11,53]. Likewise, our group has observed increased MCP-1 gene expression and secretion from 3T3-L1 murine adipocytes stimulated with low-dose LPS [43] to mimic in vivo circulating levels in obesity [83,109,254]. Further, the cross-talk between co-cultured murine adipocytes and splenic immune cells, representative of cells of the SVF within AT, has been shown to increase MCP-1 secretion [81], which our group has confirmed in unstimulated co-cultures of murine 3T3-L1 adipocytes and RAW 264.7 macrophages [44], as well as in LPS-stimulated co-cultures of murine 3T3-L1 adipocytes with either splenic CD11b^+^ macrophages [45], CD8^+^ T cells [46,47], or CD4^+^ T cells [49]. Thus, adipocyte-immune cell cross-talk may play a crucial role in the recruitment of macrophages to obese AT and the development of AT and systemic inflammation and IR [14,75,258].

AT production of MCP-1 is induced by stimuli that are reported to be elevated in the obese state, including LPS and FFA [147]. For instance, in healthy humans acutely administered LPS to mimic metabolic endotoxemia, AT production and circulating levels of MCP-1 increased prior to the development of systemic IR [85]. Further, MCP-1 synthesis and secretion was upregulated in PA- and LPS-treated human and 3T3-L1 murine adipocytes in vitro [116,147,260], but blunted by antagonizing TLR4 signaling or NF-κΒ activity [52,71] (discussed in Section 6.1). MCP-1 is also an insulin-responsive gene that remains sensitive in an insulin resistant state, as demonstrated in vitro in 3T3-L1 adipocytes induced to be insulin resistant, and in vivo in ob/ob mice [261]. In turn, MCP-1 further contributes to the development of IR as MCP-1 treatment was shown to impair 3T3-L1 adipocyte insulin-stimulated glucose uptake and the expression of several adipogenic genes, including PPARγ [261].

The significance of MCP-1 in the pathology of obesity-induced metabolic dysfunctions has been further demonstrated in HFD-fed MCP-1^−/−^ and CCR2^−/−^ mice. In brief, both knockout models were partially protected from the HFD-induced increase in adiposity and exhibited reduced AT macrophage accumulation and inflammatory adipokine production [258,262]. Further, MCP-1/CCR2-deficiency increased adiponectin expression, and improved systemic glucose homeostasis and insulin sensitivity; an effect that was mirrored by acute CCR2 antagonism in mice with established HFD-induced obesity [262]. Taken together, the metabolic endotoxemia and hyperinsulinemia that are characteristic of the obese phenotype may contribute to AT inflammation through, in part, the macrophage chemotactic function of adipocyte-derived MCP-1.

### 5.2. Interleukin-6

Circulating levels of the IL-6 are positively correlated with adiposity, circulating FFA, and IR in humans [263,264], and accordingly, increased circulating IL-6 is predictive of the development of T2D [265]. Approximately 15–35% of systemic IL-6 is secreted by AT [266], and an in vitro comparison suggested that obese visceral AT secretes more IL-6 than subcutaneous [263]. Further, IL-6 concentrations in the interstitial fluid of AT were reported to be 100-fold higher compared to circulating levels in the same participants [267], highlighting the significance of AT IL-6 secretion and its local action within obese AT.

LPS stimulates AT-derived IL-6 production [83,85] as demonstrated by the dose-dependent increase in IL-6 gene expression and secretion from human [116,145] and 3T3-L1 murine adipocytes in vitro [52,260]. Further, our group confirmed these findings in 3T3-L1 adipocytes using a low dose of LPS [43] to mimic in vivo circulating levels in obesity [83,109,254]. However, adipocytes secrete approximately 10% of total AT-derived IL-6 [263]; thus, the cells of the SVF, including M1-polarized macrophages, are considered to be the primary cellular source [11,126,268]. Nonetheless, adipocytes express the IL-6 receptor (IL-6R) [263,267], suggesting a role for IL-6 in the cross-talk between adipocytes and immune cells within AT, as confirmed in co-cultured adipocytes and a mixed population of SVF cells [81], and in co-cultured adipocytes with either CD11b^+^ macrophages [44,45], CD8^+^ T cells [46,47], or CD4^+^ T cells [49].

Upon binding IL-6, adipocyte IL-6R activates Janus kinase (JAK) family members, leading to the activation of transcription factors of the STAT family (reviewed in [269]) (shown in Figure 2). Adipocyte IL-6-signaling, in general, leads to dysregulated adipokine production and impaired insulin action. For instance, in vitro treatment with IL-6 increased 3T3-L1 murine adipocyte IL-6 synthesis and secretion in a feedforward manner [270], and decreased expression of the insulin-sensitizing adipokine, adiponectin [267]. IL-6 also induces its negative regulator, suppressor of cytokine signaling (SOCS)3, which has been shown to interfere with insulin receptor substrate (IRS)-1 and IRS-2 in 3T3-L1 murine adipocytes [271]. Accordingly, AT IL-6 was inversely correlated with insulin-stimulated adipocyte glucose uptake in vitro [264], which coincides with reduced IRS-1, glucose transporter 4, and PPARγ gene expression in cultured adipocytes treated with IL-6 [267,270,272,273]. Thus, IL-6-mediated adipocyte dysfunction and adipokine secretion may potentiate the cross-talk between adipocytes and immune cells within AT to contribute to the maintenance of chronic low-grade inflammation in obesity.

### 5.3. Tumor Necrosis Factor-α

TNF-α is a potent inflammatory adipokine that is overexpressed in human [274,275] and rodent [22,37,276] obese AT and, likewise, circulating levels of TNF-α are increased in obese humans [277] and correlate with markers of IR [278]. TNF-α is synthesized as a 26-kDa transmembrane monomer that undergoes proteolytic cleavage to yield a 17-kDa soluble TNF-α molecule; both of these are biologically active and are increased in human obese versus lean AT [279]. Characteristics of the obese phenotype, including metabolic endotoxemia, induce AT synthesis and secretion of TNF-α as evidenced by acute LPS administration in healthy humans [85]. Similarly, acute TNF-α infusion in healthy humans has been shown to induce systemic IR [280]. The significance of TNF-α in the pathology of obesity-induced metabolic dysfunctions has been further demonstrated in genetic and HFD-induced rodent models of obesity wherein TNF-α knockout or neutralization attenuated the development of systemic IR [276,281].

TNF-α expression is higher in obese visceral versus subcutaneous AT, and in cells of the SVF compared to adipocytes [21,65,275]. While TNF-α gene expression is increased in adipocytes isolated from obese versus lean humans [53], and in cultured human [116] and 3T3-L1 murine adipocytes treated with LPS [146], there are conflicting reports [145]. Additionally, M1-polarized macrophages within AT are suggested to be the primary cellular source compared to adipocytes, although further study is needed [21,65]. Nonetheless, adipocytes express TNF-α receptor (TNFR)1 and TNFR2 [275], suggesting a role for TNF-α in the cross-talk between adipocytes and immune cells within AT. The ligand-binding extracellular domains of TNFR1/2 are highly homologous, unlike the intracellular domains, which activate different signaling pathways (reviewed in [282]). The majority of evidence suggests that TNFR1 mediates the effects of TNF-α on AT dysfunction [275,283,284,285]. Indeed, neutralization of TNFR1 but not TNFR2 down-regulated expression of inflammatory adipokines in human adipocytes cultured in SVF-conditioned media [275]. Nonetheless, TNFR2 is suggested to cooperate with TNFR1 to regulate TNF-α signaling in chronic inflammatory conditions [282] such as obesity, and accordingly, only TNFR2 gene expression is reported to increase in obese versus lean human adipocytes [275,285].

Adipocyte TNF-α signaling is reported to induce inflammatory adipokine production and lipolysis via, in part, an NF-κΒ-dependent mechanism [65,69] (shown in Figure 2) and, further, TNF-α impairs insulin action by inhibiting the normal tyrosine phosphorylation of IRS-1 [284,286]. We and others have demonstrated that TNF-α secretion increases in co-cultured murine 3T3-L1 adipocytes and RAW 264.7 macrophages [44], coinciding with increased adipocyte gene expression of MCP-1, IL-6 and TNF-α, as well as increased FFA release in a similar model [65]. In turn, the adipocyte-derived SFA, PA, stimulated macrophage TNF-α gene expression [65], creating a vicious cycle that promotes AT dysfunction. Further, these inflammatory effects of adipocyte-macrophage cross-talk were determined to be independent of cell-cell contact [44,65]. Importantly, the increase in adipocyte inflammatory adipokine production and lipolysis were attenuated to a similar degree by both a TNF-α neutralizing antibody and a NF-κΒ inhibitor [65,69], which is consistent with findings in human adipocytes cultured in SVF-conditioned media [275]. Related to this, our group has shown that TNF-α gene expression and protein secretion increase in LPS-stimulated murine 3T3-L1 adipocytes co-cultured with either splenic CD11b^+^ macrophages [45], CD8^+^ T cells [46,47], or CD4^+^ T cells [49]. Interestingly, we have also shown that LPS-stimulated adipocyte-CD8^+^ T cell inflammatory cross-talk and ensuing M1 macrophage polarization and adipocyte dysfunction are attenuated by TNF-α neutralization [47,48]. In summary, TNF-α induces adipocyte lipolysis and regulates NF-κΒ activity to initiate a vicious cycle between adipocytes and immune cells that is central to the development of AT inflammation in obesity.

### 5.4. Interleukin-1β

IL-1β is a potent inflammatory adipokine that is overexpressed in human [11,53,207] and rodent [53,207,287] obese AT, and increased circulating levels are predictive of the development of T2D [265]. Compared to subcutaneous AT, obese visceral AT expresses more IL-1β, as well as the IL-1 cell membrane receptor (IL-1R), which reportedly coincides with increased AT accumulation of M1-polarized macrophage and CD8^+^ T cells [207,287,288]. Accordingly, cells of the SVF, particularly M1-polarized macrophages, are suggested to be the primary source of IL-1β within AT, although human and murine adipocyte IL-1β synthesis and secretion is markedly increased in obese versus lean AT [53,207,288]. Indeed, our group has demonstrated increased IL-1β gene expression in 3T3-L1 murine adipocytes stimulated with low-dose LPS [43] to mimic in vivo circulating levels in obesity [83,109,254]. Regardless of the cellular source, AT production of IL-1β is dependent upon the activation of NF-кB [289]; hence, IL-1β gene expression is increased in cultured human and 3T3-L1 murine adipocytes and macrophages stimulated with LPS, PA and TNF-α [53,73,116,290]. Subsequently, IL-1β is synthesized as an inert pro-protein and is cleaved by caspase-1, a cysteine protease domain of the NLRP3 inflammasome, to yield the mature, bioactive form of IL-1β in response to various obesity-induced intracellular stressors, such as ROS accumulation (reviewed in [291,292]) (shown in Figure 2; discussed in Section 6.3).

AT-derived IL-1β acts in an autocrine or paracrine fashion to contribute to obese AT inflammation and dysfunction, and accordingly, ablation of IL-1β signaling in IL-1R knockout mice attenuated the HFD-induced AT and systemic IR [287]. As a ligand for the IL-1R, which also mediates NF-кB activity [293], IL-1β has been shown to stimulate inflammatory adipokine synthesis, impair insulin-stimulated glucose uptake, and induce lipolysis in cultured human and 3T3-L1 murine adipocytes [294,295]. Thus, it is conceivable that IL-1β contributes to the inflammatory cross-talk between adipocytes and immune cells that ultimately impairs AT function. Indeed, our group has shown that LPS-stimulated 3T3-L1 murine adipocyte-specific IL-1β expression increases in co-culture with splenic CD11b^+^ macrophages [45]. Further, administration of an IL-1β neutralizing antibody blunted the expression and secretion of inflammatory adipokines, FFA release, and markers of IR in human adipocytes cultured in macrophage-conditioned media [163]. Also, our group has demonstrated that increased IL-1β gene expression coincides with increased secretion of other inflammatory adipokines in LPS-stimulated, co-cultured murine 3T3-L1 adipocytes with either splenic CD8^+^ T cells [46,47] or CD4^+^ T cells [49]. Interestingly, IL-1β and TNF-α have been shown to synergistically increase AT NF-кB activity and inflammatory adipokine production ex vivo [287]. Accordingly, TNF-α markedly increased 3T3-L1 murine adipocyte IL-1β gene expression and protein secretion [290] yet, in turn, TNF-α secretion was reduced in 3T3-L1 adipocytes co-cultured with macrophages derived from IL-1R knockout mice, which coincided with improved adipocyte insulin-stimulated glucose uptake [287]. Taken together, IL-1β contributes to the chronic low-grade inflammatory state that is characteristic of obese AT by impairing adipocyte function and regulating NF-кB activity to exacerbate adipokine dysregulation.

### 5.5. Inflammatory Adipokine Modulation by n-3 Polyunsaturated Fatty Acids in Obesity

In vitro investigations have demonstrated the anti-inflammatory effects of LC *n*-3 PUFA (EPA and DHA) on adipokine secretion (shown in Figure 2), however, some studies have shown that DHA is more potent than EPA in both the absence and presence of LPS [35,44,165]. The ability of dietary LC *n*-3 PUFA to modulate adipokines in the presence of LPS is significant in light of the contribution of metabolic endotoxemia to AT inflammation in obesity. Both LC *n*-3 PUFA (EPA and DHA) have been shown to attenuate both adipocyte (unstimulated and LPS-stimulated) [34,36,37,39,40,43,296,297] and macrophage [35,37,38,42] inflammatory adipokine secretion. Furthermore, our group has demonstrated reduced inflammatory adipokine secretion from co-cultured murine 3T3-L1 adipocytes and RAW264.7 macrophages treated with EPA or DHA or both [44], as well as from LPS-stimulated co-cultures of murine 3T3-L1 adipocytes with FO (i.e., LC *n*-3 PUFA)-enriched splenic CD11b^+^ macrophages [45], CD8^+^ T cells [46,47], or CD4^+^ T cells [49]. Likewise, LC *n*-3 PUFA supplementation has consistently been shown to reduce visceral AT production of inflammatory adipokines in rodent models of both genetic and diet-induced obesity [41,176,177,179,180,183,194,298].

In humans, LC *n*-3 PUFA have been shown to improve several metabolic risk factors, namely blood lipid levels [299]; however, the effects on circulating adipokines (i.e., cytokines) are less clear as circulating levels are not always reflective of the local AT concentrations. Instead, circulating CRP has emerged as a leading clinical inflammatory biomarker because of its association with habitual inflammatory status [209,210]. However, the effects of LC *n*-3 PUFA on any inflammatory markers in overweight/obese individuals are unclear; thus, determination of the minimum effective dosage of LC *n*-3 PUFA to modulate such critical aspects of the obese phenotype are limited, perhaps owing to differences between study designs, the inflammatory biomarkers analyzed, the duration of LC *n*-3 PUFA intervention, as well as the source (i.e., dietary sources versus supplements) and dose of LC *n*-3 PUFA interventions. In this connection, a range of LC *n*-3 PUFA intake levels (for example, 0.6–6.0 g/day EPA + DHA) from dietary sources and supplements has been shown to result in a range of outcomes in circulating inflammatory mediators in overweight/obese individuals, as reviewed by our research group [30,31] and others [28,32,33]. For instance, in randomized controlled interventions, dietary intake of LC *n*-3 PUFA reduced circulating levels of CRP and IL-6 in obese men [300], and improved circulating levels of CRP and IL-6 but not TNF-α in overweight women [301]. Further, purified DHA supplementation reduced circulating CRP, IL-6 and TNF-α levels in obese men and women, whereas EPA supplementation only reduced IL-6 [302]. Despite these findings, other studies report no association between LC *n*-3 PUFA intake and circulating inflammatory cytokines [303,304,305,306,307,308]. Thus, given the controversy, circulating levels of the aforementioned inflammatory cytokines may not be the optimal or reproducible primary endpoint in human studies to assess the efficacy of LC *n*-3 PUFA supplementation in improving aspects of the obese phenotype.

### 5.6. Adiponectin and Leptin

Adiponectin and leptin are two AT-derived adipokines whose functions tend to oppose one another in respecitvely attenuating or promoting obese AT inflammatory dysfunction (reviewed in [309]). In obesity, AT synthesis and circulating levels of adiponectin are reduced [310,311,312,313] such that adiponectin concentrations are inversely correlated with adiposity [312]. Adiponectin circulates in different oligomeric forms of trimeric, hexameric, or high molecular weight (HMW) [314], wherein the levels of the HMW isoform correlates most closely with systemic insulin sensitivity [315]. Thus, adiponectin exerts insulin-sensitizing effects and improves lipid metabolism in adipocytes and in peripheral tissues such as liver and skeletal muscle (reviewed in [316,317]). Accordingly, adiponectin deficiency was shown to induce IR in a rodent model, whereas its overexpression improved insulin sensitivity and glucose tolerance [318]. Similarly, in a rodent model of genetic obesity, overexpression of adiponectin reversed many characteristic components of the obese phenotype, resulting in improved glucose and lipid metabolic parameters, decreased circulating inflammatory adipokine levels, and reduced macrophage AT infiltration compared to obese littermates [313].

Adipocyte and/or visceral AT adiponectin expression and secretion is downregulated by inflammatory adipokines whose expression is increased within obese AT [272,294,319,320]. Specifically, the suppressive effects of TNF-α on adiponectin secretion from adipocytes can be partially recovered by a JNK inhibitor [320]. Conversely, adiponectin stimulation has been shown to suppress LPS-induced inflammatory adipokine production in adipocytes [321] via inhibition of NF-κB [322] and upregulation of PPARγ expression [323]; beneficial anti-inflammatory effects that are attenuated in obesity. Adiponectin antagonizes inflammatory adipokine expression by inhibiting NF-κB activation [324,325,326,327], and by stimulating anti-inflammatory IL-10 secretion from macrophages [328,329,330,331], which express the adiponectin receptor (AdipoR1/R2) and are responsive to adiponectin signaling [332]. Functionally, adiponectin has been shown to suppress M1 macrophage activation and production of inflammatory adipokines, in favour of promoting the polarization of M2 macrophages [323,331,332,333]. Additionally, adiponectin has been shown to influence dendritic cell function by decreasing the expression of co-stimulatory molecules (CD80/CD86) that resulted in reduced CD4^+^ T cell proliferation and increased FOXP3^+^ Treg expansion [334]; effects that could be beneficial in obese AT given the reduced tissue abundance of Tregs [22,23,128,206], although further study is required.

Obesity is also characterized by sustained elevated circulating levels of the adipokine leptin, which is positively associated with body fat mass [335,336,337]. While many cell types express the leptin receptor (Ob-Rb), including adipocytes, myocytes and hepatocytes, it is also expressed by immune cells such as T cells and macrophages, suggesting an immunomodulatory role for leptin in obese AT [211,338]. In this connection, leptin exerts inflammatory effects by promoting the secretion of inflammatory adipokines, namely TNF-α and IL-6 [338,339]. In turn, inflammatory stimuli such as LPS and other inflammatory adipokines stimulate leptin expression in AT, thereby creating a feedforward loop that perpetuates the low-grade chronic inflammatory phenotype that characterizes obese AT [340,341,342,343]. In this connection, leptin has also been shown to promote macrophage activation, proliferation, and enhanced phagocytic activity and secretion of inflammatory adipokines [338,339,344,345]. Leptin also induces T cell proliferative responses by polarizing CD4^+^ T cells towards the Th1 IFN-γ-secreting subset [211,346] and inhibiting Treg proliferation [347,348], whose cellular abundance in obese AT declines relative to AT mass [22,23,128,129,130]. Taken together, leptin contributes to the inflammatory microenvironment of obese AT.

### 5.7. Adiponectin and Leptin Modulation by n-3 Polyunsaturated Fatty Acids in Obesity

Dietary LC *n*-3 PUFA have been shown to increase circulating levels of adiponectin in obesity in both humans [305,349,350,351,352] and rodent models [179,180,351,353,354], thereby attenuating the obesity-associated reduction in adiponectin [310,311,312,313]. Our group has shown that EPA and DHA reduces the gene expression of M1 macrophage markers via an adiponectin-dependent mechanism [44,45]. Furthermore, LC *n*-3 PUFA function as activators of PPARγ [355] and, as such, LC *n*-3 PUFA have been shown to upregulate adiponectin production in adipocytes via a PPARγ-dependent mechanism [356,357,358,359]. Furthermore, PPARγ antagonizes NF-κB nuclear activity through a trans-repression mechanism, thereby decreasing the expression of NF-κB responsive genes [360], including inflammatory adipokines, as elegantly demonstrated in vivo by macrophage-specific deletion of PPARγ, which highlighted the critical role of PPARγ in the regulation of macrophage polarization (promoting the M2 phenotype and reducing M1), as well as AT and systemic inflammation and metabolic dysfunction [361].

Dietary LC *n*-3 PUFA have also been shown to reduce AT gene expression and/or circulating levels of leptin in overweight and obese humans [362,363] and rodents [178,364,365], wherein EPA has been shown to be more effective versus DHA [366]. Although the specific mechanisms through which LC *n*-3 PUFA exert these effects are undetermined, the ability to reduce leptin production within dysfunctional obese AT likely contributes to the LC *n*-3 PUFA-mediated reduction in inflammatory adipokine secretion and changes in AT macrophage and T cell function. Collectively, these data demonstrate the critical effect of LC *n*-3 PUFA on AT adipokine production that ultimately attenuates both the metabolic and inflammatory dysfunction that characterizes obese AT.

## 6. Cell Signaling Mechanisms Regulating Adipokine Production in Obese Adipose Tissue

### 6.1. TLR2/4 and NF-κB Regulation of Adipokines

Within AT, adipocytes and immune cells both express several signaling receptors including PRR, TLR2 and TLR4 [51,52,115], which respond to pathogen-associated molecular patterns (PAMPs) and contribute to the development of AT inflammation and IR in the obese state (reviewed in [54]). LPS is a well-established ligand for TLR4 within AT [52,83] and SFA (e.g., PA) are reported to be ligands for both TLR2 and TLR4 [66,68,69,117], although controversy exists [40,71]. A consequence of TLR2/4 stimulation is the activation of the NF-κB transcription factor complex [52] (shown in Figure 2). Specifically, the stimulation of TLR2/4 induces myeloid differentiation primary-response protein (MyD)88/interleukin-1 receptor-associated kinase (IRAK)1 signaling, which leads to the phosphorylation and activation of transforming growth factor-β activated kinase (TAK)1 by promoting its association with the TAK1 binding protein (TAB)1 [367]. Active TAK1 promotes NF-κB activation by phosphorylating and activating the inhibitor of κB (IκB) kinase (IKK) complex to downregulate IκB and allow NF-κB to translocate to the nucleus [367]. In turn, NF-κB regulates the expression of inflammatory and chemotactic adipokines [52,143,368], and thus, plays a pivotal role in the innate and adaptive immune responses within obese AT. Accordingly, the expression of TLR2 and TLR4, as well as the activity of NF-κB, are increased in obese and type 2 diabetic humans [109,369,370], and in rodent models of HFD-induced obesity [66,142,371,372].

The significance of TLR2/4 signaling in the pathology of obesity-induced metabolic dysfunctions has been demonstrated in vitro and in knockout mouse models fed a HFD. For instance, a substantial amount of evidence supports that LPS induces NF-κB activity and the production of inflammatory adipokines, as well as impairs insulin signaling in cultured human [109,116,369] and 3T3-L1 murine adipocytes [43,65,71]; all of which was inhibited by co-treatment with an antibody against TLR4 [52]. TLR2 and TLR4-dependent NF-κB activity and inflammatory adipokine production have also been demonstrated in adipocytes and macrophages treated with the SFA, PA [64,65,66,67,68,69,70,71,72]. Interestingly, in vitro TLR4 signaling was demonstrated to increase TLR2 expression in adipocytes [66], and co-culture of adipocytes with TLR4-deficient macrophages significantly attenuated inflammatory adipokine gene expression and adipocyte lipolysis [69], suggesting that TLR2/4 signaling contributes to the inflammatory cross-talk between adipocytes and immune cells within obese AT. Indeed, our group has demonstrated that LPS increases inflammatory adipokine expression and secretion in 3T3-L1 murine adipocytes alone [43] and in co-culture with splenic CD11b^+^ macrophages [45], CD8^+^ T cells [46,47], or CD4^+^ T cells [49]. In vivo, TLR2 and TLR4 knockout rodent models were protected against the HFD-induced increase in visceral AT mass, NF-κB activity and gene expression of inflammatory adipokines, which coincided with reduced circulating levels of MCP-1 and ensuing AT accumulation of M1-polarized macrophages [66,117,142]. Further, the same rodent models were protected again HFD-induced IR [66,117,141]; an effect that was also demonstrated in TLR4 knockout mice infused with lipids [66] and infused with LPS to induce metabolic endotoxemia [83]. Taken together, TLR2 and TLR4 play a crucial role in modulating adipokines in response to circulating LPS and SFA, and therefore represent potential targets for dietary intervention during development of the obese phenotype.

### 6.2. TLR2/4 and NF-κB Modulation by n-3 Polyunsaturated Fatty Acids

During the progression of obesity, LC *n*-3 PUFA antagonize AT inflammation by antagonizing LPS- and SFA-induced TLR2/4 signaling in adipocytes and immune cells. Specifically, LC *n*-3 PUFA are suggested to block the ligand binding sites of TLR2 and TLR4, as demonstrated in cultured RAW 264.7 murine macrophages wherein the LPS- and SFA-induced activation of NF-κB was inhibited by EPA and DHA [64,372]. Likewise, our group has shown that EPA and DHA blunt LPS-induced inflammatory adipokine gene expression and specifically MCP-1 secretion [43].

The incorporation of LC *n*-3 PUFA (EPA and DHA) into cell membranes disrupts the formation of lipid rafts that are necessary for TLR4 signaling [122,123,373,374]. In cultured macrophages, DHA inhibited the LPS- and SFA-induced recruitment of TLR4 and MyD88 to lipid raft fractions, which coincided with reduced ROS accumulation and NF-κB activity [123]. Similarly, our group has shown that NF-κB activity and/or inflammatory adipokine production is blunted in LPS-stimulated co-cultures of murine 3T3-L1 adipocytes and FO (i.e., LC *n*-3 PUFA)-enriched murine splenic CD11b^+^ macrophages [45] or CD8^+^ T cells [46,47]. It is conceivable that the TLR4 response to LPS was perturbed in LC *n*-3 PUFA-enriched immune cell-adipocyte co-cultures in a lipid raft-dependent manner, but the underlying mechanisms remain unknown. TLR2 activation may also be dependent upon lipid raft formation to promote TLR2 dimerization with cell membrane-bound TLR1 or TLR6 [54], and accordingly, DHA was reported to inhibit TLR2 signaling and subsequent inflammatory adipokine production in adipocytes and macrophages in vitro [37,72,375].

### 6.3. GPR120-Dependent Regulation of Adipokines

GPR120 is highly expressed in adipocytes and macrophages and plays a pivotal role in the maintenance of metabolic homeostasis [37]. Although body weight and AT mass were unaffected, GPR120 knockout mice exhibit impaired insulin sensitivity compared to wildtype [37]. Likewise, in a HFD-fed obese rodent model, GPR120 knockout increased body weight and AT mass, as well as AT M1 macrophage accumulation, consistent with a greater degree of systemic IR compared to WT rodents [376]. In turn, macrophage-conditioned media has been shown to inhibit GPR120 expression in human adipocytes [377], as has the classically M1 macrophage-derived TNF-α and IL-1β [378]. Interestingly, GPR120 expression is increased in human obese versus lean visceral AT, yet its dysfunction is associated with the development of the obese phenotype [376].

GPR120 expression is dependent on functional PPARγ and vice versa [378,379], and both of these respond to *n*-3 PUFA [37,42,359,380,381]. The *n*-6 PUFA, arachidonic acid (AA), is reported to induce GPR120 signaling, though is a less potent ligand compared to EPA and DHA [382], and SFA exert no effect on GPR120 signaling [37]. Ligand-stimulated GPR120 promotes the association of GPR120 and β-arrestin2 (βarr2), an adaptor protein that mediates GPR120 internalization and signaling [37] (shown in Figure 2). The GPR120- βarr2 complex then interrupts TLR2/4 signaling by associating with TAB1 to block the association between TAB1 and TAK1 and, therefore, block TAK1 phosphorylation/activation and downstream NF-κB activation [37,42,383]. Thus, GPR120 may represent a link between dietary LC *n*-3 PUFA and the modulation of inflammatory adipokines in obesity.

### 6.4. GPR120 Modulation by n-3 Polyunsaturated Fatty Acids

The role of GPR120 in mediating the effects of dietary LC *n*-3 PUFA has primarily been interpreted from EPA and DHA-treated adipocytes and macrophages in vitro [37,42,378,380,381,383], and in GPR120 knockout rodent models fed a HFD enriched with *n*-3 PUFA [37,376]. For instance, EPA and DHA attenuated the LPS- and TNF-α-induced gene expression and secretion of inflammatory adipokines in cultured macrophages via a GPR120- and βarr2-dependent mechanism [37,42]. Specifically, the anti-inflammatory actions of EPA and DHA in vitro mimicked those induced by a selective GPR120 agonist, GW9508, and were lost in macrophages isolated from GPR120 and βarr2 knockout rodent models [37,42]. Similarly, our group has shown that the anti-inflammatory effects of DHA are mimicked by GW9508 in 3T3-L1 murine adipocytes [43] wherein other work has shown that GPR120-agonism mitigates NF-κB activation [383]. Ultimately, GPR120 is reported to regulate adipocyte function as DHA increased basal and insulin-stimulated glucose uptake in 3T3-L1 murine adipocytes, but the effect was abrogated by GPR120 knockdown [37].

In HFD-fed rodents, the insulin-sensitizing effects of EPA and DHA supplementation were lost as a result of GPR120 knockout, which coincided with increased M1 macrophage accumulation and inflammatory adipokine expression in obese visceral AT [37,376]. Importantly, the change in MCP-1 expression was specific to adipocytes, and both MCP-1 and adipocyte-conditioned media promoted macrophage chemotaxis in vitro [37]; an effect that was blunted by macrophage pre-treatment with DHA, but not in macrophages isolated from GPR120 knockout mice [37], suggesting the potential for GPR120 to mediate the anti-inflammatory action of LC *n*-3 PUFA on adipocyte-immune cell cross-talk within obese AT. Further, our group has shown that EPA and DHA attenuate the expression and secretion of inflammatory adipokines in co-cultured murine 3T3-L1 adipocytes and RAW264.7 macrophages [44], and likewise in LPS-stimulated co-cultures of murine 3T3-L1 adipocytes and FO (i.e., LC *n*-3 PUFA)-enriched splenic CD11b^+^ macrophages [45], CD8^+^ T cells [46,47], or CD4^+^ T cells [49]. While the underlying mechanisms remain unknown, it is conceivable that GPR120 signaling negatively regulated inflammatory signaling in these co-culture models, although further study is required.

### 6.5. NLRP3 Inflammasome-Dependent Regulation of Adipokines

During the progression of obesity, the NLRP3 inflammasome regulates the innate immune response within AT (reviewed in [291,292]). Inflammasomes are multi-protein complexes comprised of a danger-sensing intracellular PRR from the family of NLR, such as NLRP3; the cysteine protease, caspase-1; and the adaptor protein, apoptosis-associated speck-like protein containing a caspase-recruitment domain (PYCARD) (reviewed in [291,292]). The NLRP3 inflammasome assembles in response to many stimuli including endogenous obesity-induced metabolites (reviewed in [291,292]), such as the accumulation of ROS [384,385,386] (shown in Figure 2). Next, active caspase-1 cleaves pro-IL-1β and pro-IL-18 to produce their mature isoforms [291,292]. In turn, IL-1β, specifically, impairs AT insulin signaling [163,287,295] and, hence, increased circulating levels of IL-1β are predictive of the development of T2D [265].

In humans, adipocyte but not SVF expression of NLPR3 inflammasome components and activation of the caspase-1 domain correlate with adiposity [53], suggesting that hypertrophic and dysfunctional adipocytes dominate the NLRP3 inflammasome-mediated response in obesity. Accordingly, the expression and activation of the NLRP3 inflammasome is increased in obese versus lean [53,203,207,387], and visceral versus subcutaneous AT [288], which coincides with increased IL-1β secretion form obese visceral AT [207,288]. Further, NLRP3 inflammasome (i.e., caspase-1) activity correlates with increased M1 macrophage and CD8^+^ T cell accumulation in visceral AT of obese humans [207,288]. Related to this, our group has shown that the gene expression of NLRP3 inflammasome components, IL-1β production, and/or caspase-1 activity are increased in co-cultures of murine 3T3-L1 adipocytes and CD11b^+^ macrophages [45], CD8^+^ T cells [46,47], or CD4^+^ T cells [49], highlighting the significance of adipocyte-immune cell cross-talk in the activation of the NLRP3 inflammasome. Accordingly, NLRP3 knockout mice were protected against the HFD-induced immune cell (e.g., macrophages, CD8^+^ T cells) infiltration into visceral obese AT, which coincided with reduced AT inflammation and improved systemic insulin sensitivity [203,388].

NLRP3 inflammasome activity is regulated by two signals; an initial priming signal to induce the gene expression of NLRP3 and pro-IL-1β, and a second signal to activate the multi-protein complex to promote caspase-1 activity [291,292]. Priming of the NLRP3 inflammasome is potently induced by inflammatory signals that activate the NF-κB transcription factor to regulate NLRP3 and IL-1β expression, such as inflammatory adipokines or those transmitted via TLR2/4 (e.g., LPS or SFA) to induce MyD88/IRAK1 signaling [72,143,144,389]. In addition to priming, the MyD88/IRAK1 signaling axis directly links TLR2/4 signaling to activation of the NLRP3 inflammasome [390], perhaps, in part, via a ROS-dependent mechanism as SFA (e.g., PA) and LPS have been shown to prime and activate the NLRP3 inflammasome in cultured adipocytes and macrophages, yet the effects were blunted by antioxidant pre-treatment [39,72,73,74]. Thus, ROS accumulation may be a crucial intermediate in TLR2/4-mediated NLRP3 inflammasome activity and IL-1β secretion in obese AT. Importantly, secreted IL-1β can act in an autocrine or paracrine fashion as a ligand for IL-1R, which also induces MyD88/IRAK1 signaling [293] and has been demonstrated to prime the NLRP3 inflammasome in cultured adipocytes [53]. Therefore, the NLRP3 inflammasome mediates a vicious cycle within obese AT that contributes to adipokine dysregulation and systemic IR.

### 6.6. NLRP3 Inflammasome Modulation by n-3 Polyunsaturated Fatty Acids

Recent evidence suggests that dietary fatty acids can modulate NLRP3 inflammasome activity (reviewed in [391]), which is suppressed by LC *n*-3 PUFA in particular. For instance, DHA supplementation reduced AT NLRP3 inflammasome (i.e., caspase-1) activity and IL-1β production in a HFD-induced obese rodent model, which coincided with improved systemic insulin sensitivity to a similar degree that was observed in NLRP3 knockout rodents consuming the same diet [42]. Interestingly, the effects of DHA were lost in NLRP3 knockout rodents, suggesting that the anti-inflammatory and insulin-sensitizing effects of DHA during a HFD are dependent upon the inhibition of NLRP3 inflammasome activity [42]. Unfortunately though, to our knowledge, this is the only in vivo investigation into the ability of dietary LC *n*-3 PUFA to modulate the NLRP3 inflammasome in obesity. However, related to this, our group established an in vitro co-culture model of obese AT and showed that LC *n*-3 PUFA decrease NLRP3 inflammasome priming, IL-1β production, and/or caspase-1 activity in LPS-stimulated co-cultures of murine 3T3-L1 adipocytes and CD11b^+^ macrophages [45], CD8^+^ T cells [46,47], or CD4^+^ T cells [49].

Other in vitro evidence supporting LC *n*-3 PUFA-mediated inhibition of the NLRP3 inflammasome suggests mechanisms that overlap with their inhibition of TLR2/4 signaling [37,42]. For instance, in murine bone marrow-derived macrophages, pre-treatment with EPA, DHA and, to a lesser extent, ALA inhibited the LPS-induced priming of the NLRP3 inflammasome and activation of the caspase-1 domain, which coincided with reduced IL-1β secretion [42]. Interestingly, the ability of EPA and DHA to inhibit NLRP3 inflammasome activation was not dependent upon their enzymatic metabolism to lipid mediators (e.g., resolvins), but was, in part, dependent upon a GPR120-mediated interaction between βarr2 and NLRP3 [42]. However, since GPR120 signaling was only partially responsible for the LC *n*-3 PUFA-mediated inhibition of NLRP3 inflammasome activity [42], another mechanism must be involved. Accordingly, DHA has been shown to disrupt the PA-induced recruitment of MyD88 to lipid rafts, which inhibited TLR2/4 activation, downstream ROS accumulation, and IL-1β secretion from human macrophages [72].

## 7. Conclusions

Obese AT inflammation and dysfunction is sustained via multiple interrelated mechanisms that integrate the effects of altered immune cell AT infiltration and altered cellular abundance of inflammatory subsets [12,13,21,22,23,24,25], with cross-talk via paracrine interactions with adipocytes to stimulate the secretion of a panel of inflammatory adipokines that collectively influence local AT and systemic metabolic function [11,15,16,19,60,79,80]. Interestingly, we have outlined both the immune cell- and adipocyte-mediated mechanisms through which LC *n*-3 PUFA impact AT immune cell polarization and function via critical cell signaling pathways within AT to modulate inflammatory adipokine secretion as well as local (i.e., AT) and systemic metabolic function, thereby improving these critical aspects of the obese phenotype. Further research is required to explore these LC *n*-3 PUFA-mediated mechanisms to improve obese AT dysfunction and to determine how other dietary fatty acids can influence these cellular processes. Additionally, determining the effective dosage of LC *n*-3 PUFA intake (either through dietary or supplemental sources) as well as that of other dietary fatty acids, namely plant-derived *n*-3 PUFA (i.e., ALA) represent other potential future directions. Collectively, the AT response to dietary LC *n*-3 PUFA may provide a strategy to mitigate obesity-associated AT inflammation prior to the development of systemic IR and T2D.
